# Close temporal coupling of neuronal activity and tissue oxygen responses in rodent whisker barrel cortex

**DOI:** 10.1111/j.1460-9568.2011.07927.x

**Published:** 2011-12

**Authors:** Jennifer Li, Diego S Bravo, A Louise Upton, Gary Gilmour, Mark D Tricklebank, Marianne Fillenz, Chris Martin, John P Lowry, David M Bannerman, Stephen B McHugh

**Affiliations:** 1 Lilly Centre for Cognitive Neuroscience, Discovery Biology, Lilly Research CentreLilly UK, Windlesham, Surrey, UK; 2 Department of Physiology, Anatomy and Genetics, University of OxfordOxford, UK; 3 Gray Institute for Radiation Oncology and Biology, Department of Oncology, University of Oxford, Churchill HospitalOxford, UK; 4 Department of Chemistry, National University of IrelandMaynooth, Co. Kildare, Ireland; 5 Department of Experimental Psychology, University of Oxford9 South Parks Road, Oxford, UK

**Keywords:** amperometry, barrel cortex, hemodynamic response, local field potential, tissue oxygen

## Abstract

Neuronal activity elicits metabolic and vascular responses, during which oxygen is first consumed and then supplied to the tissue via an increase in cerebral blood flow. Understanding the spatial and temporal dynamics of blood and tissue oxygen (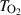) responses following neuronal activity is crucial for understanding the physiological basis of functional neuroimaging signals. However, our knowledge is limited because previous 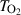 measurements have been made at low temporal resolution (>100 ms). Here we recorded 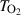 at high temporal resolution (1 ms), simultaneously with co-localized field potentials, at several cortical depths from the whisker region of the somatosensory cortex in anaesthetized rats and mice. Stimulation of the whiskers produced rapid, laminar-specific changes in 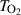. Positive 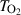 responses (i.e. increases) were observed in the superficial layers within 50 ms of stimulus onset, faster than previously reported. Negative 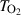 responses (i.e. decreases) were observed in the deeper layers, with maximal amplitude in layer IV, within 40 ms of stimulus onset. The amplitude of the negative, but not the positive, 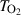 response correlated with local field potential amplitude. Disruption of neurovascular coupling, via nitric oxide synthase inhibition, abolished positive 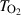 responses to whisker stimulation in the superficial layers and increased negative 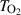 responses in all layers. Our data show that 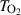 responses occur rapidly following neuronal activity and are laminar dependent.

## Introduction

Functional magnetic resonance imaging (fMRI) is now the dominant methodology for investigating human brain function but it does not measure neuronal activity directly. Instead, fMRI uses secondary correlates to infer neuronal activity, e.g. the blood-oxygen-level-dependent (BOLD) signal. Understanding the complex processes linking neuronal activity with downstream metabolic and hemodynamic changes is therefore imperative if we are to understand what neuroimaging signals represent and their spatial and temporal limitations.

Measuring neuronal activity concomitantly with BOLD in the scanner presents major technical challenges (Goense *et al.*, 2010). An alternative approach employed by several laboratories is to measure tissue oxygen (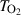) as a proxy for BOLD, using the rationale that 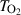 changes are driven by the same physiological mechanisms as changes in the BOLD signal (Thompson *et al.*, 2003; Offenhauser *et al.*, 2005; Masamoto *et al.*, 2008; Lowry *et al.*, 2010). These studies have significantly advanced our knowledge of the neurometabolic and neurovascular mechanisms that underlie BOLD. However, to date, 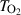 has been measured at relatively low temporal resolution (typically > 100 ms per data point), largely for technical reasons. Thus, although neuronal activity takes place within milliseconds, we have no knowledge of 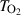 responses at a millisecond timescale. To address this issue, here we measured 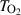 at high temporal resolution (1 ms) concomitantly with local field potentials (LFPs) in the whisker-barrel pathway of anaesthetized rats and mice.

High temporal resolution 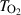 measurements were achieved using constant potential amperometry at uncovered carbon paste electrodes (CPEs). Previous studies have measured 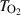 with gold or platinum electrodes, housed within a protective membrane that prevents electrode deterioration and subsequent loss of oxygen (O_2_) sensitivity. However, the membrane also increases the diffusion time for O_2_ to reach the electrode surface and thus limits the temporal resolution (for discussion see Bolger *et al.*, 2011). CPEs do not require this protective membrane and their temporal resolution is limited only by O_2_ diffusion kinetics (<1 ms).

We have previously used this approach to study hippocampal 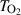 responses in freely-moving rats during cognitive and emotional tasks (McHugh *et al.*, 2011) but, because the stimuli (e.g. spatial cues) were not time-locked to the 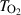 recordings, the temporal parameters of the 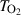 response remain unknown. Here we used single electrical pulses or mechanical deflections of the whiskers to elicit neuronal and 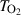 responses in the barrel cortex. The whisker-barrel sensory pathway is an ideal network for this approach because it has a topographical and columnar spatial organization, and well-defined vasculature, such that somatic stimulation produces discrete and reproducible responses within each cortical layer. Moreover, neuronal responses differ between cortical layers, with the earliest firing units and the largest amplitude LFPs found in layer IV (Armstrong-James & Fox, 1987; Di *et al.*, 1990). Here we investigated the laminar specificity of 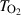 responses and LFPs by recording sequentially at multiple depths within the barrel cortex. In addition, we investigated the relationship between the magnitude of LFPs and 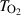 responses by varying the intensity of whisker stimulation. Finally, we investigated the effects of disrupting neurovascular coupling on 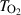 responses by inhibiting neuronal nitric oxide synthase (nNOS) with 7-nitroindazole (7-NI).

## Materials and methods

### Constant potential amperometry for tissue oxygen

Changes in 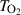 were recorded using constant potential amperometry at CPEs as described previously (Lowry *et al.*, 1997; Bolger *et al.*, 2011). In this technique, the CPE is held at a constant potential (−650 mV relative to a reference electrode) using a potentiostat (‘Biostat’; ACM Instruments, Cumbria, UK or Electrochemical and Medical Systems Ltd, Newbury, UK). The application of this potential causes the electrochemical reduction of O_2_ at the surface of the CPE, which induces an electrical current that is measured by the potentiostat. The availability of O_2_ for this two-step reaction (O_2_ + 2H^+^ + 2e^−^ → H_2_O_2_; H_2_O_2_ + 2H^+^ + 2e^−^ → 2H_2_O) is determined by the local 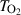 concentration. Therefore, when 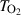 increases (e.g. following an increase in cerebral blood flow (CBF) or when there is a decrease in O_2_ utilization during constant CBF), the current increases linearly, and when 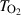 decreases (e.g. when O_2_ utilization is greater than CBF, or there is a relative decrease in CBF), the current decreases linearly. In this way, changes in local 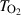 concentration directly produce proportional changes in the amperometric signal (Hitchman, 1978).

### Electrode construction

The CPEs were made from 8T (200 μm bare, 270 μm coated diameter; Experiment 1) or 5T (125 μm bare, 177 μm coated diameter; Experiment 2) Teflon®-coated silver wire (Advent Research Materials, Suffolk, UK). The Teflon insulation was slid along the wire to create a 2 mm deep cavity that was packed with carbon paste. The Teflon coating on the CPEs was flush with the tip of the electrode such that the active part of the electrode was a flat disk with diameter 250 μm (area: 0.05 mm^2^) in Experiment 1 or 125 μm (area: 0.01 mm^2^) in Experiment 2. The carbon paste was prepared by mixing 2.8 g of carbon powder (Sigma-Aldrich, St Louis, MO, USA, catalogue no. 282863) and 1.0 mL of silicone oil (Sigma-Aldrich, catalogue no. 17563-3) (O’ Neill *et al.*, 1982). LFP electrodes were made from 5T Teflon-coated silver wire. Co-localized LFP and 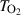 recordings were achieved by twisting the LFP electrode around the CPE, making a double electrode with the active tips level in the dorsal–ventral plane. The distance between the electrodes in the medial–lateral/anterior–posterior planes was approximately 150 μm. Reference electrodes for the 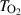 recordings were made from 8T Teflon®-coated silver wire. All wire electrodes were soldered to gold connectors (E363/0; Plastics One, Roanoke, VA, USA). Skull screws served as auxiliary electrodes (O_2_ and LFP circuits) and reference electrodes (LFP circuit).

### Electrode calibration

The linear response of CPEs to changes in O_2_ concentration was confirmed by *in vitro* calibration, using a three-electrode glass electrochemical cell (BASi C3 cell stand, Bioanalytical Systems, USA ) containing 15 mL phosphate-buffered saline (pH 7.4) with a silver/silver chloride reference electrode and a platinum auxiliary electrode (Bioanalytical Systems). Calibrations were performed in nitrogen (N_2_)-purged, air-saturated, and O_2_-saturated solutions with O_2_ concentrations of 0, 240, and 1200 μm, respectively. Calibration coefficients for each CPE were calculated by plotting a line of best fit through the three data points by least squares linear regression and taking the slope as the coefficient in nA/μm [full details of this procedure can be found in Bolger *et al.* (2011)]. Mean (±SEM) coefficients were 1.42 (±0.14) nA/μm for the 250 μm diameter CPEs used in Experiment 1 and 0.75 (±0.02) nA/μm for the 125 μm diameter CPEs used in Experiment 2. Raw 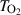 signals (in nA) from each CPE were multiplied by their coefficient to produce a calibrated 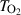 signal in μm.

### Electrode properties

The characterization of CPEs and constant potential amperometry (at −650 mV) for measuring 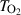 has recently been published in detail (Bolger *et al.*, 2011). In summary, the 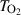 signal shows high sensitivity (0.5−1.5 nA/μm), low detection limits (∼0.1 μm) and near-linear responses (*r* > 0.9) to changes in O_2_ concentration. *In vitro*, the 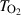 signal responds rapidly (within 1 s) to changes in O_2_ concentration and shows low stirring sensitivity (∼3–4%). Moreover, the 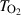 signal is insensitive to changes in pH, temperature, or ionic concentration (Ca^2+^and Mg^2+^) within the normal physiological range. The O_2_ consumption of the electrodes is approximately 1.1 nmol/h, which is small compared with the 40–80 μm O_2_ concentration in the extracellular fluid (Erecinska & Silver, 2001). The amperometric detection of O_2_ is based on the electrochemical reduction of O_2_ and therefore the 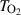 signal is free from interference from oxidizable analytes within the extracellular fluid. Moreover, the 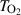 signal shows negligible interference from other electroactive species present in brain tissue (e.g. ascorbic acid, dopamine, serotonin, homovanillic acid, 5-hydroxyindoleacetic acid, 3,4-dihydroxyphenylacetic acid, l-tyrosine, l-cysteine, l-tryptophan, l-glutathione, dehydroascorbic acid, and uric acid).

### Experimental design overview

Two experiments were carried out, one in rats and one in mice. Apart from species, there were several methodological differences between the experiments (Experiment 1 vs. Experiment 2): sex (male vs. female), anaesthetic (urethane vs. halothane), method of ventilation (spontaneous breathing vs. artificial ventilation), angle of electrode penetration into cortex (vertical vs. perpendicular to cortex), CPE active surface diameter (250 vs. 125 μm), and method of whisker stimulation (electrical pulse to whisker pad vs. mechanical deflection of whiskers). The objective of both experiments was the same, i.e. to investigate the laminar specificity of 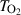 and neuronal responses in the somatosensory cortex at high temporal resolution. The same pattern of results was observed in both experiments, despite these methodological differences, testifying to the generality of the data.

## Subjects

Experiment 1 used seven adult male Sprague-Dawley rats (280–350 g at the time of surgery) and Experiment 2 used 11 adult female C57/BL6 mice (17–30 g at the time of surgery). All procedures were performed in accordance with the UK Animals (Scientific Procedures) Act 1986.

### Surgery

#### Experiment 1

Rats were anaesthetized with 1.5 mL 25% urethane solution (∼2 g/kg, i.p.) and placed in a stereotaxic frame with the head level between bregma and lambda. Lack of withdrawal reflexes was tested throughout the experiment and additional doses of urethane were given if necessary to ensure a stable level of anaesthesia. The temperature of the animal was maintained at 37 °C using a rectal probe and a thermostatically-controlled heating blanket (Harvard Apparatus, MA, USA). An incision was made in the scalp and the periosteum resected. Five holes were drilled in the skull and the underlying dura was pierced with a hypodermic needle. One hole allowed the insertion of a double LFP/CPE into the somatosensory cortex (anterior-posterior: −2.5 mm; medial-lateral: +5.5 mm from bregma). LFP and 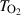 responses were investigated at several cortical depths from 0 mm (cortical surface) to 2 mm below the cortical surface. A second hole drilled into the contralateral hemisphere (approximate anterior-posterior: −2.0 mm; medial-lateral: +3.0 mm from bregma) allowed insertion of the 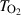 reference electrode into the cortex. Skull screws were inserted into the remaining holes to act as an auxiliary electrode for the 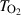 circuit and as reference and auxiliary electrodes for the LFP circuit. Rats were breathing spontaneously during surgery and all subsequent recording.

#### Experiment 2

Mice were first anaesthetized using Hypnorm/Hypnovel (10 μL/g, i.p.; Hypnorm; Janssen Pharmaceutica: fentanyl citrate 0.315 mg/mL; fluanisone 10 mg/mL, Hypnovel; Roche: midazolam 5 mg/mL) and local anaesthetic was then applied to the throat (EMLA cream; APP Pharmaceuticals, 5% emulsion containing 2.5% each of lidocaine and prilocaine) and a tracheotomy was performed. The mice were then connected to a ventilator (Mini Vent Type 845; Hugo Sachs Elektronik, Germany) with the respiration set at 130 strokes/min and 175 μL/stroke. Anaesthesia was maintained using halothane (1.5–2%) in a mixture of O_2_ and NO_2_. The mouse was then placed in a stereotaxic frame with the head level between bregma and lambda, an incision was made in the scalp and the periosteum resected. A hole was drilled into the skull to allow the insertion of a double LFP/CPE into the somatosensory cortex (AP: −1.5 mm; ML: −3.0 mm from bregma). The centre of this craniotomy was over the whisker barrel cortex corresponding to the D2 whisker region. LFP and 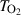 responses were investigated at five cortical depths from 0.05 mm (layer I) to 0.85 mm (layer VI) below the cortical surface in 0.2 mm steps. A second hole was drilled in the contralateral hemisphere to allow insertion of the reference electrode (approx AP: −1.0 mm; ML: +1.5 mm from bregma). An auxiliary electrode was placed in the scalp. During stereotaxic surgery and subsequent recordings, the heart rate was monitored via two electrocardiogram wires inserted into the armpits and connected to a Cardiotachometer (CT 100; CWE Inc., PA, USA); end tidal CO_2_ was also monitored (Micro Cap Star CO_2_ analyzer; CWE Inc.) and typically maintained at ∼4% during recordings. Throughout the experiment, body temperature was thermostatically controlled at 37 °C. Mice were artificially ventilated throughout stereotaxic surgery and during all subsequent recordings.

### Data recording

In both experiments, LFP electrodes were connected to a differential amplifier (Harvard Apparatus) and the signal was sent to an analogue/digital converter (Micro 1401; CED, Cambridge, UK) and onto a PC running either Signal 2.10 (Experiment 1) or Spike2 (Experiment 2) software (CED). LFPs were recorded at 15 kHz (Experiment 1) or 5 kHz (Experiment 2). 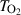 measurements were made using the potentiostat, which contained a custom-built pre-amplifier. The output of the potentiostat was connected to an analogue/digital converter (Experiment 1: Powerlab 8/30, AD Instruments, Oxon, UK; Experiment 2: Micro 1401; CED) and then onto a PC running digital acquisition software (Experiment 1: Chart v5, AD Instruments; Experiment 2: Spike2; CED). 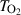 responses were recorded continuously at 1 kHz (Experiment 1) or 5 kHz (Experiment 2). 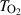 and LFP data were downsampled to 1 kHz for analysis. Once electrodes were implanted into the brain, the potential (−650 mV) was applied and the CPEs were left to settle for 30 min before any experiments began.

### Whisker stimulation

In Experiment 1, electrical stimulation of the rat whisker pad was used to elicit LFPs and 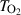 responses. Two needle electrodes were attached to a stimulator (Isolated Pulse Stimulator; A-M Systems, USA) and placed 2 mm subcutaneously in a posterior direction between rows A/B and C/D of the whisker pad, contralateral to the barrel cortex recording site. Square-wave pulse stimulations of 0.3 ms duration were administered every 10 s. The timing and intensity of stimuli were controlled by Signal 2.10 software via the Micro 1401 analogue/digital converter (CED). In Experiment 2, mechanical stimulation of the whiskers was used to elicit neuronal and 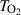 responses. Approximately 10 whiskers, surrounding the D2 whisker, were inserted into a small metal cannula that was attached to a piezo-electric wafer (multilayer piezo bender actuator, model PL140.11, Physik Instrumente, Germany). Applying a voltage (using the piezo driver, E-650; Physik Instrumente) caused a deformation of the wafer that, in turn, produced a controlled movement of the cannula and hence the whiskers. Sinusoidal pulse stimulations of 20 ms duration were administered every 1 s and were controlled by Spike2 v5 software via the Micro 1401 analogue/digital converter (CED).

### Procedures

#### In vitro and in vivo control experiments

Control experiments were performed to demonstrate (i) that the 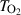 signal was free from electrical interference and (ii) that it detected changes in O_2_ availability.

First, the susceptibility of the 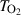 signal to changes in electrical potential was investigated *in vitro* using a three-electrode electrochemical cell (Bioanalytical Systems) containing 15 mL phosphate-buffered saline (pH 7.4) with a Ag/AgCl reference electrode, a Pt auxiliary electrode, and a CPE for O_2_ detection. The polarizing potential (−650 mV) was applied to the CPE and the phosphate-buffered saline was bubbled with air (∼20% O_2_) to establish a baseline O_2_ signal. Changes in 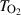 (Δ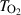) were measured with reference to this baseline. Trains of electrical stimuli (1–100 Hz, +5 V, 0.5 ms) were then administered via a bipolar stimulating electrode placed into the phosphate-buffered saline to simulate electrical neuronal activity. This stimulation protocol was performed six times. Electrical stimulation of the cell had no effect on the 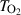 signal (Supporting Information Fig. S1). Therefore, as this level of stimulation is more than an order of magnitude greater than that typically seen *in vivo*, it is extremely unlikely that changes in electrical potential within the brain tissue affected the 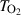 signals in the whisker stimulation experiments described below.

**Fig. 1:**
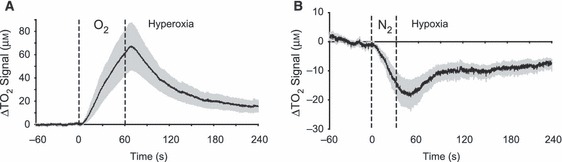
CPEs recording the partial pressure of 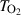 in the whisker-barrel region of the somatosensory cortex respond to changes in systemic O_2_ availability *in vivo* (*n* = 6 rats). The black line shows the mean 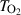 and the grey line shows ±SEM. (A) Inhalation of O_2_ (hyperoxia) increased the 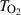 signal. (B) Inhalation of N_2_ (hypoxia) decreased the 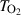 signal.

The second control experiment investigated the sensitivity of the 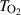 signal to changes in O_2_ availability and was performed in anaesthetized rats. With the 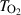 electrodes positioned in the barrel cortex, mild hyperoxia and hypoxia were induced by applying gaseous O_2_ (BOC Medical, Manchester, UK) or N_2_ (BOC Gases, Guildford, UK), respectively, to the snout of the anaesthetized rats. Polyurethane tubing, connected to the appropriate gas cylinder, was held approximately 2 cm from the snout and the gas delivered for either 60 s (O_2_) or 30 s (N_2_) at a flow rate of ∼1 L/min. O_2_ inhalation increased the 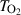 signal by 33.0 ± 12.0 μm after 30 s (61.7 ± 18.2 μm after 60 s), whereas N_2_ inhalation decreased the 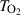 signal by 16.2 ± 3.1 μm after 30 s (Fig. 1). A paired *t*-test revealed that the 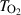 signal was significantly higher after O_2_ than N_2_ inhalation [*n* = 6 rats; *t*(5) = 3.3; *P* = 0.02]. This demonstrates that the 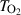 signal is highly sensitive to changes in O_2_ availability *in vivo*.

### Whisker stimulation experiments

#### Experiment 1

##### Depth profile

**Fig. 2:**
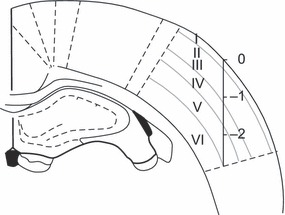
Reconstruction of recording sites in rat whisker barrel cortex (Experiment 1) showing the vertical angle of electrode insertion. Recordings were made at 10 depths from 0 mm (cortical surface) to −1.8 mm (approximately layer V). The scale bar is in mm. [Figure adapted with permission from Elsevier. The figure was published in The Rat Brain in Stereotaxic Coordinates, Paxinos, G. & Watson, C., ©Academic Press (1998)].

To investigate the laminar specificity of LFP and 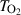 responses, we created a depth profile by recording sequentially at 10 cortical depths. The order was counterbalanced such that, in some animals, recordings were made at the dorsal-most site first and in others at the ventral-most site first. Using the arm of the stereotaxic frame, the dual CPE/LFP electrode was carefully lowered/raised in 0.2 mm steps from 0 mm (brain surface) to 1.8 mm below the brain surface (*n* = 3), or from −1.8 to 0 mm (*n* = 4), with 10 stimulations given at each depth (1.2 mA, 0.3 ms pulses). Note that, as the Teflon coating was flush with the electrode tip, the active surface of the CPE was entirely within brain tissue even at 0 mm. Electrode penetrations were made such that the electrodes entered the brain at a vertical angle (see Fig. 2). Due to this angle, we estimated the most ventral recording site (1.8 mm below the brain surface) to be in layer V.

##### Effects of stimulus intensity on local field potential and tissue O_2_ responses

The depth profile was used to determine the site of maximum LFP amplitude, which, based on previous studies (Di *et al.*, 1990), we assumed to be in layer IV. LFP and 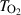 responses were then recorded to a range of stimulus intensities (0.5–3.0 mA in 0.5 mA steps; 0.3 ms pulses in all cases) with the electrodes fixed in the same position in the cortex throughout (i.e. layer IV). Ten stimulations were given at each intensity level.

##### Effects of local anaesthetic into ipsilateral or contralateral whisker pad

To demonstrate that 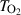 responses (and LFPs) were due to neuronal transmission in the whisker-barrel pathway and not a stimulation or other electrical artefact, local anaesthetic was injected into either the ipsilateral or contralateral whisker pad. If 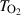 responses were caused by a stimulation artefact, then injection into either the ipsilateral or contralateral whisker pad should have no effect. However, if 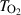 responses are dependent upon neuronal transmission then contralateral injections should reduce 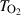 responses (and LFPs), whereas ipsilateral injections should have little effect because of decussation of the sensory pathway. With the electrodes positioned in layer IV (based on the maximum evoked LFP amplitude), we established a pre-injection baseline for LFP and 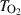 responses by giving 10 whisker stimulations (1.2 mA, 0.3 ms). Lignocaine hydrochloride (1%; 10 mg in 1 mL 0.9% NaCl) was then injected (0.1 mL, SC) into the ipsilateral whisker pad and 10 stimulations were administered. A second lignocaine injection was then made into the contralateral whisker pad followed by 10 whisker stimulations.

##### Effects of sustained whisker stimulation

To investigate the effect of sustained stimulation, we applied a 10 Hz train to the whisker pad for 40 s, with the electrodes positioned in layer IV. (Note that this procedure was performed before the local anaesthetic injection).

#### Experiment 2

The primary aims of Experiment 2 were to (i) replicate the findings of Experiment 1 in a different species and with mechanical movements of the whiskers rather than electrical stimulation; and (ii) investigate whether the rapid positive 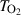 responses seen in the superficial layers during Experiment 1 were affected by disrupting neurovascular coupling. To achieve aim (ii), a subset of mice (*n* = 4) were injected with 7-NI (50 mg/kg, i.p.; Sigma-Aldrich, catalogue no. N7778; 5 mg/mL in peanut oil vehicle, Sigma-Aldrich, catalogue no. P2144) approximately 45 min before recording began. Previous studies have shown that this dose of 7-NI reduces stimulus-evoked CBF by 50–70% without reducing the field potential amplitude (Iadecola *et al.*, 1996; Yang *et al.*, 1999, 2000). The drug was sonicated in vehicle for 5 min immediately before injection. Three mice were injected with vehicle only (0.2–0.3 mL peanut oil) and four mice served as uninjected controls. There were no differences between the vehicle and uninjected groups in LFP amplitudes [*t*_5_ = 1.4; *P* = 0.19] or 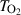 amplitudes [*t*_5_ = 1.4, *P* = 0.22] and they were combined into a single control group.

##### Depth profile

**Fig. 3:**
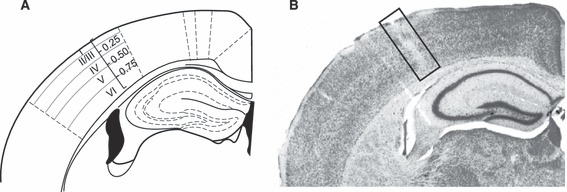
Reconstruction (A) and representative photomicrograph (B) showing recording positions from the mouse barrel cortex (Experiment 2). Note that the angle of electrode insertion is perpendicular to the cortical lamina. Recordings were made at five depths, corresponding to layers I, II/III, IV, V, and VI. The scale bar is in mm. [Figure adapted with permission from Elsevier. The figure was published in The Mouse Brain in Stereotaxic Coordinates, Paxinos, G. & Franklin, K.B.J., ©Academic Press (2001)].

The LFP and 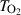 responses were investigated in 7-NI-treated and control mice at five cortical depths in the barrel cortex (0.05, 0.25, 0.45, 0.65, and 0.85 mm below the brain surface), corresponding to layers I, II/III, IV, V, and VI, respectively. Note that in Experiment 2 the stereotaxic arm was positioned at an angle such that the electrodes penetrated perpendicular to the cortical surface (see Fig. 3). The dual CPE/LFP electrode was carefully lowered from layer I (0.05 mm) to layer VI (0.85 mm) in 0.2 mm steps (control group, *n* = 3; 7-NI group, *n* = 2) or raised from layer VI (0.85 mm) to layer I (0.05 mm) in 0.2 mm steps (control group, *n* = 4; 7-NI group, *n* = 2). One hundred stimulations were administered at each depth. During recording, the preparation was insulated with silicon oil (Sigma).

##### Perfusion and histology

After the experiment, a subset of mice from Experiment 2 were perfused for histological determination of the electrode placements (Fig. 3). Mice were injected with 0.1 mL of pentobarbital (Euthatal, 200 mg/mL) and perfused transcardially with physiological saline (0.9% NaCl) and then formol–saline (10% formalin in 0.9% NaCl). The brains were removed and preserved in formol–saline. They were then transferred to a 30% sucrose–formalin solution for 24 h and frozen. Coronal sections (50 μm) were cut on a freezing microtome and stained with cresyl violet to enable visualization of the electrode tracks.

### Signal processing and analysis

**Fig. 4:**
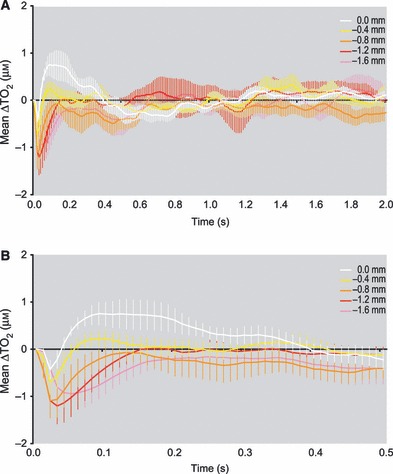
Mean (±SEM) 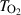 responses at five cortical depths in response to electrical stimulation of the whisker pad (1.2 mA, 0.3 ms) in rats (*n* = 7). (A) There was no evidence of stimulus-induced changes in 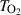 beyond the first 1 s after stimulus onset. (B) Same data as in A but showing 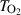 responses during 0.5 s after stimulus onset.

This study was primarily concerned with rapid and transient responses, and our analyses are therefore mostly restricted to 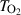 signals and LFPs occurring within the first 1 s after stimulus onset. Stimulus-induced changes to single pulses or deflections were typically not observed beyond 1 s (see Fig. 4). Stimulus-induced LFP and 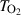 responses were calculated as the mean response for 10 stimulations in Experiment 1 (i.e. at each cortical depth, stimulus intensity, or injection condition) or 100 stimulations in Experiment 2 (i.e. at each cortical depth). Changes in 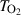 (Δ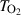) were calculated by subtracting a baseline (the mean 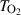 signal in the 50 ms before stimulus onset) from the stimulus-induced 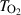 signals.

#### Local field potential metrics

The LFP amplitude was calculated as the maximum-to-minimum value occurring 4–50 ms (Experiment 1) or 4–100 ms (Experiment 2) after stimulus onset (i.e. LFP amplitude = |maximum amplitude–minimum amplitude|). The difference in time windows for the two experiments reflected the different LFPs elicited by electrical (Experiment 1) and mechanical (Experiment 2) stimulation. The slope (b) of the LFP was calculated as the least squares linear regression through *y*-values occurring 4–9 ms after stimulus onset, where *y* = *bx* + *a*.

#### Tissue O_2_ metrics

For 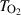 responses we calculated maximum-to-minimum amplitude (|maximum amplitude–minimum amplitude|), maximum amplitude (i.e. for positive 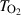 responses), and minimum amplitude (i.e. for negative 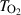 responses) occurring within the 100 ms after stimulus onset. The slope (b) of the 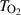 response was calculated as the least squares linear regression through *y*-values occurring 0–20 ms after stimulus onset. When negative, the slope of the 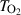 response gave a measure of the rate of O_2_ decrease in the tissue (e.g. from increased utilization or decreased CBF). Positive and negative areas under the curve (AUCs) for 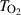 responses were calculated separately and gave measures of cumulative increases in 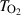 (positive AUC) or decreases in 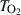 (negative AUC) over time. This measure is useful because 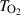 responses exhibit inter-subject variability in their precise temporal kinetics and timepoint-to-timepoint averaging can obscure important differences that are captured by AUC. The time windows for AUC were 0–200 ms (Experiment 1), 0–500 or 0–900 ms (Experiment 2), where 0 ms denotes stimulus onset. These time windows were chosen based on the duration of positive and negative 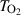 responses in the two experiments. AUCs were calculated using the trapezoidal method in Prism v4 (Graphpad Software, CA, USA), which treats a timeseries curve as a series of connected XY points that form trapeziums with width ΔX (i.e. a timebin) and heights *Y*1 and *Y*2, with area Δ*X* × ½ (*Y*1 + *Y*2). The onset latency of 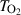 responses was defined as a signal change greater than two SDs of baseline fluctuations (i.e. during the 50 ms pre-stimulus period) that was sustained for at least 5 ms. Positive (i.e. above baseline) and negative (i.e. below baseline) 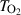 onset latencies were determined separately for each cortical depth (note that not all rats had both positive and negative responses above baseline at every cortical depth). To compare the temporal characteristics of 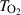 and LFP responses, we also calculated the time-to-peak (e.g. negative peak) amplitude for each signal.

#### Statistical analyses

Statistical analyses were performed in SPSS (IL, USA) or SigmaStat (SPSS) using paired *t*-tests, Pearson correlation (*r*), or anova using a general linear model (presented in the form [*A*_k_ × *B*_m_ × *S*_n_] where *A* is a factor with *k* levels, *B* is a factor with *m* levels and *n* is the number of subjects in the analysis). *Post-hoc* tests were performed using the Newman–Keuls or least significant difference method. Data are presented as arithmetic mean ± 1 SEM, unless otherwise stated. For clarity, error bars have been omitted from some figures.

## Results

### Experiment 1: laminar tissue O_2_ and local field potential responses in rat whisker barrel cortex

#### Local field potentials and tissue O_2_ responses are laminar specific

**Fig. 5:**
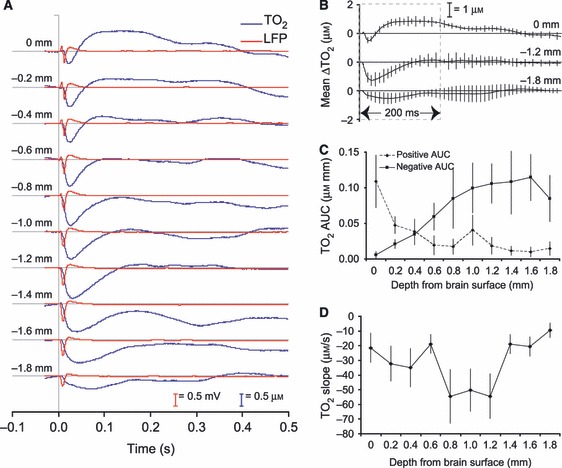
Depth profile of LFP and 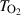 responses in the rat whisker barrel cortex (*n* = 7 rats) following electrical stimulation of the whisker pad (1.2 mA, 0.3 ms). (A) Mean LFP (red trace) and 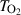 (blue trace) responses at 10 cortical depths (0 to −1.8 mm below the cortical surface) for 30 ms before and 500 ms after stimulus onset. (B) Mean (±SEM) 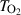 responses at 0, −1.2 and −1.8 mm below the brain surface (same data as blue traces in A). (C) Mean (±SEM) positive and negative AUCs for 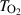 responses (0–200 ms after stimulus onset). (D). Mean (±SEM) slope of 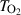 responses (0–20 ms after stimulus onset).

The LFPs and 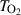 responses were measured at 10 depths in the rat barrel cortex in response to whisker pad stimulation. LFPs were reproducible within a given cortical lamina but varied between laminae (Fig. 5A, red traces). LFPs exhibited a depth profile consistent with that of previous studies (Di *et al.*, 1990). At the cortical surface, the LFP consisted of a fast positive/negative complex. At progressively deeper penetrations, the early positive component reduced in amplitude and the negative component came earlier and increased in amplitude. The largest amplitude negative deflections were observed at 1.2 ± 0.1 mm below the brain surface, which we estimate to be in layer IV, with a peak negative value at 12.1 ± 1.2 ms after stimulus onset. The amplitude of the negative deflection was reduced at recording sites ventral to 1.2 mm.

The 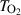 responses were also laminar specific but had different shapes and temporal dynamics compared with LFPs recorded at the same depth (Fig. 5A, blue traces). At the cortical surface (0 mm), there was a small initial decrease in 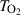 that was followed by a much larger overshoot above baseline (Fig. 5A,B, top trace). In most cases, the overshoot in 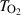 was clearly evident within 100 ms after stimulus onset and was consistently observed only in the superficial recording sites (from 0 to 0.4 mm below the brain surface, i.e. in layer I and layer II/III). At more ventral cortical depths, the 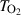 decrease became larger in amplitude (and slope), reaching a maximum at 1.2 mm below the brain surface (i.e. at the same site as the largest amplitude LFPs, in layer IV). The 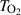 responses at this depth exhibited a negative peak at 32.7 ± 4.0 ms after stimulus onset, followed by a rapid return to baseline with little subsequent overshoot (see Fig. 5A). When the electrodes were positioned ventral to 1.2 mm, the amplitude (and slope) of the initial 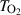 decrease became smaller, and the return to baseline became slower, but with no consistent overshoot above baseline.

To analyse the laminar differences in the 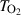 response, we calculated positive and negative AUCs for each cortical depth (Fig. 5C). Separate repeated-measures anovas were performed on the positive and negative AUCs with depth as the within-subjects factor (anova: cortical depth_10_ × *S*_7_). There was a significant effect of cortical depth on the positive AUC (*F*_9,54_ = 4.3; *P* < 0.001), with the largest positive AUC in the superficial layers. There was also a significant effect of cortical depth on the negative AUC (*F*_9,54_ = 3.7; *P* < 0.001), which was greatest in the deeper layers (Fig. 5C). Thus, the timecourse, magnitude, and direction of 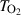 responses were dependent on the cortical depth of the 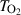 electrode. We performed a further anova on the effect of cortical depth on the slope of the 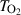 response, which revealed a significant effect of cortical depth (*F*_9,54_ = 5.4; *P* < 0.001), with steeper (negative) gradients at recording sites 0.8–1.2 mm below the brain surface, i.e. around the same depth that the maximum LFPs were recorded (Fig. 5D). Thus, the rate of O_2_ consumption was greatest at the site of maximum neuronal activity and decreased markedly outside this region.

#### Positive and negative tissue O_2_ onset latencies differ between cortical lamina

**Fig. 6:**
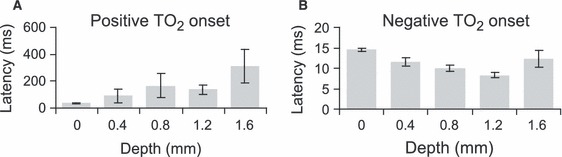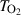 onset latencies (i.e. signal change greater than two SDs of baseline fluctuations) in the rat whisker barrel cortex for five of the 10 recording depths. Onset times for (A) positive and (B) negative 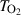 responses.

The 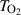 onset latencies (i.e. signal change greater than two SDs of baseline fluctuations) for five of the 10 depths are shown in Fig. 6A (positive 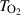 latency) and B (negative 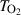 latency). Positive 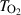 latencies were lowest at the cortical surface (36 ± 6 ms at 0 mm) and higher and more variable at deeper sites (e.g. 310 ± 123 ms at 1.6 mm). Negative 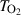 latencies were lowest in layer IV (8 ± 1 ms at 1.2 mm) and highest at the cortical surface (e.g. 14 ± 0.3 ms at 0 mm). Positive and negative 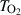 onset latencies were analysed in separate repeated-measures anovas (anova: cortical depth_5_ × *S*_7_). There was a main effect of cortical depth for positive 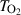 latencies (*F*_4,15_ = 4.5; *P* = 0.01), driven by the low-onset latency at 0 mm and the high-onset latency at 1.6 mm. There was also a main effect of cortical depth for negative 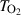 latencies (*F*_4,18_ = 4.8; *P* = 0.008), driven by the low-onset latency at 1.2 mm and the higher latencies at 0 and 1.6 mm. Thus, both positive and negative 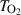 latencies differed between cortical layers.

#### Correlations between local field potential and tissue O_2_ responses

**Fig. 7:**
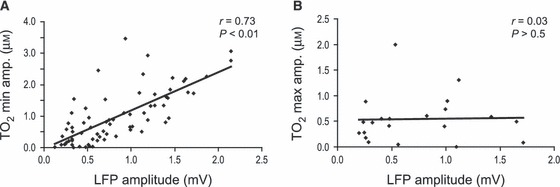
Depth profile. Relationship between LFP amplitude and 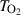 amplitude in the rat whisker barrel cortex (*n* = 7 rats). (A) Correlation between LFP amplitude and minimum (i.e. negative) 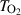 amplitude (all depths, *n* = 70 observations). (B) Correlation between LFP amplitude and maximum (i.e. positive) 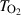 amplitude from the three most superficial cortical recording sites (i.e. where positive 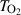 responses were consistently seen, *n* = 21 observations). In all cases, responses were evoked by electrical stimulation of the whisker pad (1.2 mA, 0.3 ms).

**Fig. 8:**
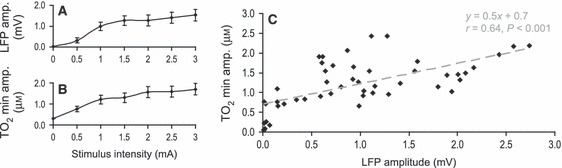
Effects of stimulus intensity on LFP and 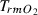 amplitude in layer IV. Input–output curves showing the effects of varying stimulus intensity (0–3 mA, 0.3 ms) on LFP amplitude (A) and the amplitude of the 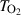 decrease (B). The correlation between LFP amplitude and negative 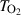 amplitude is shown in C, with the linear fit in grey (*n* = 7 rats, 49 pairs of observations).

We investigated the predictive relationship between the LFP and 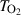 responses in two ways. First, we plotted LFP amplitude against 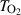 amplitude for responses recorded during the depth profile (Fig. 7). Second, we generated input–output curves for LFP and 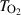 responses to a range of stimulus intensities (0–3 mA in 0.5 mA steps) when the electrodes were positioned in layer IV, and then plotted LFP amplitude against the amplitude of the 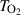 decrease (Fig. 8).

#### Negative but not positive tissue O_2_ response amplitude correlates with local field potential amplitude

For responses recorded during the depth profile, the linear correlation between LFP and 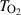 amplitudes for individual rats ranged from *r* = 0.44 to *r* = 0.91 (mean *r* = 0.67 ± 0.06). When data were combined across animals and the 10 cortical depths (*n* = 7 rats, *n* = 70 pairs of observations) there was a significant correlation between LFP amplitude and total (i.e. maximum to minimum) 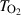 amplitude [*r* = 0.65; *t*_68_ = 7.1, *P* < 0.001] but the correlation was stronger for LFP amplitude vs. minimum 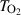 amplitude [i.e. 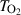 decrease, *r* = 0.73; *t*_68_ = 8.8, *P* < 0.001; Fig. 7A]. In contrast, there was no significant correlation between LFP amplitude and maximum 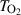 amplitude (i.e. 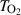 overshoot) when considering responses from the three depths (0, 0.2, 0.4 mm) that showed consistent positive 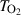 responses [*r* = 0.03; *t*_19_ = 0.1, *P* = 0.9; Fig. 7B].

#### Local field potential amplitude predicts negative tissue O_2_ amplitude in layer IV

With the electrodes positioned in layer IV, higher stimulus intensities elicited larger amplitude LFPs (predominantly the negative component) and larger amplitude 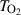 decreases (Fig. 8). Linear correlations between LFP amplitude and negative 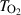 amplitudes for individual rats ranged from *r* = 0.67 to *r* = 0.98 (mean *r* = 0.87 ± 0.04). When data were combined across animals and across the seven stimulus intensities (*n* = 7 rats, *n* = 49 pairs of observations), there was a significant linear correlation [*r* = 0.64; *t*(47) = 5.7; *P* < 0.001]. These data and the correlational data from the depth profile suggest that, when evoked by single pulses, LFP amplitude is a good predictor of the size of negative but not positive 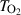 amplitude.

#### Tissue O_2_ response is not due to stimulation or recording artefact

The short latency of detectable negative and (especially) positive 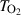 responses has, to our knowledge, never been reported before. It is important therefore to consider any methodological factors that could have influenced our results. First, could 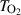 signals reflect a stimulation artefact or some other type of electrical interference from the recording equipment? This would seem unlikely because of the laminar differences in 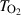 responses (i.e. if it is an artefact then it is not a uniform artefact) but we also addressed this possibility with a pharmacological manipulation. We injected local anaesthetic (lignocaine) into the whisker pads either ipsilateral or contralateral to the hemisphere containing the recording electrodes (which were positioned in layer IV). If the 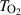 response was a stimulation (or equipment interference) artefact then injection into either whisker pad should have little or no effect as stimulation and recording parameters remained constant. Alternatively, if the 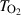 response depends upon neuronal transmission in the whisker-barrel pathway, then ipsilateral injection should have little or no effect but contralateral injection should reduce 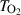 responses (and LFPs).

**Fig. 9:**
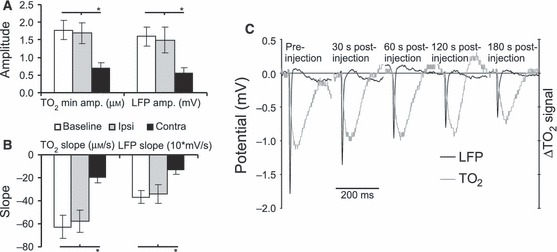
Effects of lignocaine injection (1%, 0.1 mL in 0.9% NaCl) into the rat whisker pad (*n* = 5 rats) on whisker-stimulation-evoked LFP and 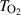 responses in layer IV. Contralateral but not ipsilateral lignocaine reduced 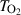 and LFP amplitude (A) and slope (B). In all cases, responses were evoked by electrical stimulation of the whisker pad (1.2 mA, 0.3 ms). *P < 0.05. (C) Gradual reduction in stimulus-evoked LFP and 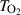 amplitude and slope following lignocaine injection into the contralateral whisker pad (representative example from one rat).

Consistent with the second hypothesis, ipsilateral lignocaine had little effect but contralateral lignocaine dramatically reduced the slope and amplitude of 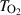 responses and the LFP (Fig. 9). Separate analyses were performed on the LFP and 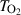 slopes and amplitudes [anova: injection condition (baseline, contralateral lignocaine, ipsilateral lignocaine)_3_ × *S*_5_]. There were significant effects of injection condition on the LFP slope (*F*_2,8_ = 13.0; *P* = 0.003) and LFP amplitude (*F*_2,8_ = 10.7; *P* = 0.005), which were lower in the contralateral condition compared with both pre-injection baseline (slope: *P* = 0.008; amplitude *P* = 0.01) and ipsilateral (slope: *P* = 0.04; amplitude *P* = 0.05) conditions. There were no differences between the ipsilateral and baseline conditions for LFP slope (*P* = 0.3) or LFP amplitude (*P* = 0.4). Similarly, there were effects of injection condition on the 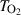 slope (*F*_2,8_ = 13.5; *P* < 0.003) and 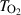 amplitude (*F*_2,8_ = 10.3; *P* = 0.006), which were lower in the contralateral condition compared with both baseline (slope: *P* = 0.01; amplitude: *P* = 0.02) and ipsilateral (slope: *P* = 0.02; amplitude: *P* = 0.04) conditions. There were no differences between the ipsilateral and baseline conditions for 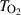 slope (*P* = 0.4) or 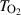 amplitude (*P* = 0.7). These results demonstrate that the observed 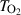 responses (and LFPs) were dependent on neuronal transmission within the whisker-barrel pathway and that 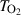 signals are not due to a stimulation artefact or electrical interference from the recording equipment.

#### Is the tissue O_2_ response contaminated by the local field potential?

Another possibility is that the 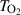 response is contaminated by, or simply reflects, the (electrical) neuronal activity inherent in the LFP. This would seem unlikely given the control experiment presented in the Materials and methods (see Supporting Information Fig. S1), where the 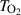 signal was unaffected by electrical activity *in vitro*, and also because the temporal dynamics (and shapes) of LFP and 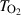 responses were quite different. For example, in the superficial layers, the LFP exhibited a positive–negative complex that reversed in polarity in the deeper layers to a negative–positive complex. In contrast, in all layers, the 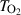 response was first negative (i.e. below baseline) and then positive (returning to or overshooting baseline; see Fig. 5). In addition, as reported earlier, the correlation between maximum-to-minimum LFP amplitude and minimum 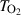 amplitude was higher than the correlation between maximum-to-minimum LFP and maximum-to-minimum 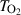 amplitude (*r* = 0.73 vs. *r* = 0.65). If the early 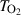 signal was a function of the LFP then one would expect a stronger correlation between the total amplitudes of the respective signals.

##### Time-to-peak measures in local field potential and tissue O_2_ response are negatively correlated

In addition, if the 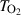 signal reflected the electrical activity of the LFP, one would expect the time-to-peak (e.g. minimum peak) values for the LFP and 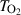 to be positively correlated. However, time-to-minimum LFP amplitude was negatively correlated with time-to-minimum 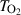 amplitude (mean for individual rats: *r* = −0.6 ± 0.1; range: *r* = −0.2 to *r* = −0.9). This is because there was a negative correlation between LFP latency and LFP amplitude (i.e. shorter latency LFPs tended to be higher amplitude; mean correlation between LFP amplitude and LFP time-to-minimum: *r* = −0.6 ± 0.2), and LFP amplitude was positively correlated with minimum 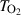 amplitude (Fig. 7A). In contrast, shorter latency time-to-minimum 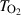 responses tended to have smaller negative 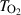 amplitude (mean correlation between minimum 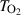 amplitude and time-to-minimum 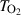 amplitude: *r* = −0.3 ± 0.2). In other words, as the negative peak of the LFP came earlier, the negative peak of the 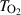 response came later. It is difficult to see how, if the 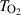 signal was simply a downsampled, filtered or skewed function of the electrical activity of the LFP, the time-to-peak latencies of the two signals could be negatively correlated. Thus, a methodological explanation for the rapid 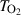 responses observed in Experiment 1 does not seem tenable.

#### Sustained stimulation leads to positive tissue O_2_ response

**Fig. 10:**
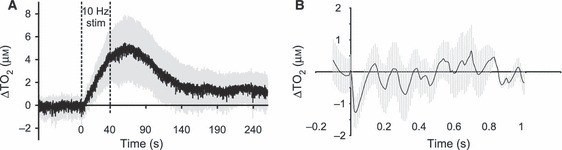
(A) Continuous whisker stimulation (10 Hz, 40 s) in layer IV elicited a slower onset and sustained 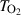 increase in layer IV of the rat barrel cortex. (B) Responses to individual whisker stimulations in the first 1 s of the stimulus train showed a decrease in 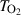 (mean 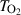 signal in black, SEM in grey; *n* = 5 rats).

To investigate 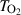 responses to sustained stimulation, we applied electrical stimulation to the whisker pad at 10 Hz for 40 s when the electrodes were positioned in layer IV (*n* = 5 rats). This elicited a substantial 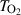 increase (Fig. 10A) lasting tens of seconds, although the response to individual stimuli of the train (e.g. during the first 1 s) was a 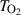 decrease (Fig. 10B) similar to that observed to single electrical pulses (cf. Fig. 5, 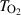 responses at −1.2 mm). Thus, sustained stimulation produced a characteristically slower-onset but positive 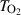 response, replicating the late-phase 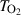 kinetics shown in previous studies (e.g. Offenhauser *et al.*, 2005).

### Experiment 2: laminar tissue O_2_ and local field potential responses in control mice vs. mice treated with nitric oxide synthase inhibitor (7-nitroindazole)

**Fig. 11:**
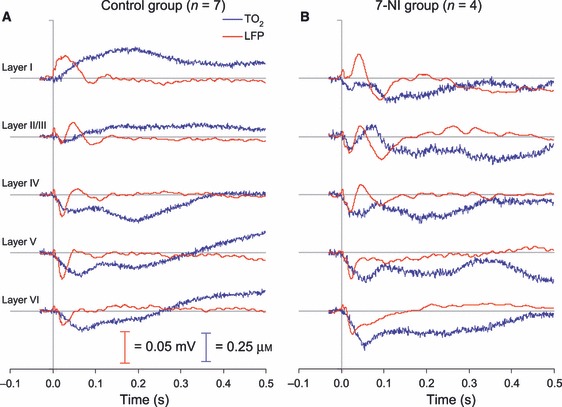
Depth profile of LFP and 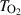 responses from the mouse whisker barrel cortex following whisker stimulation in control mice (A, *n* = 7) and mice treated with the nNOS inhibitor, 7-NI (B, *n* = 4). Recordings were made at five depths, corresponding approximately to layers I, II/III, IV, V, and VI. Mean LFPs are shown in red; mean 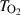 responses are shown in blue. In all cases, responses were evoked by mechanical whisker deflection (20 ms sinusoidal pulse applied to piezo-electric wafer producing a controlled movement of a cannula containing ∼10 whiskers).

Experiment 2 had two objectives: (i) to see if the short latency 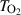 responses seen in rats in Experiment 1 were also present in mice; and (ii) if so, to see if 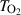 responses were affected by systemic disruption of neurovascular coupling via inhibition of nNOS. To this end, we generated depth profiles for LFP and 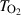 responses in the barrel cortex to whisker deflections in control mice (uninjected, *n* = 4; vehicle, *n* = 3, Fig. 11A) and mice treated with the nNOS inhibitor, 7-NI (*n* = 4, Fig. 11B).

#### No effect of 7-nitroindazole on local field potentials

The LFPs showed similar laminar profiles in controls and 7-NI-treated mice. In layer I (0.05 mm), the LFP was characterized by a positive–negative complex, which reversed in layer II/III (0.25 mm) to a negative–positive complex, and which was largely negative in the deeper layers. The largest amplitude negative deflection was recorded in layer IV (0.45 mm), with a peak negative value at 25.4 ± 2.1 ms after stimulus onset. LFPs were similar in the 7-NI-treated and control mice, and there was no evidence that 7-NI reduced LFP amplitude at any cortical depth. (Note that the differences in LFP shape, timing, and amplitude between Experiments 1 and 2 were probably due to the different whisker stimulation methods and parameters used, i.e. 0.3 ms electrical stimulation of the whisker pad in Experiment 1 vs. 20 ms mechanical deflection of the whiskers in Experiment 2.)

#### 7-nitroindazole decreases positive tissue O_2_ responses

**Fig. 12:**
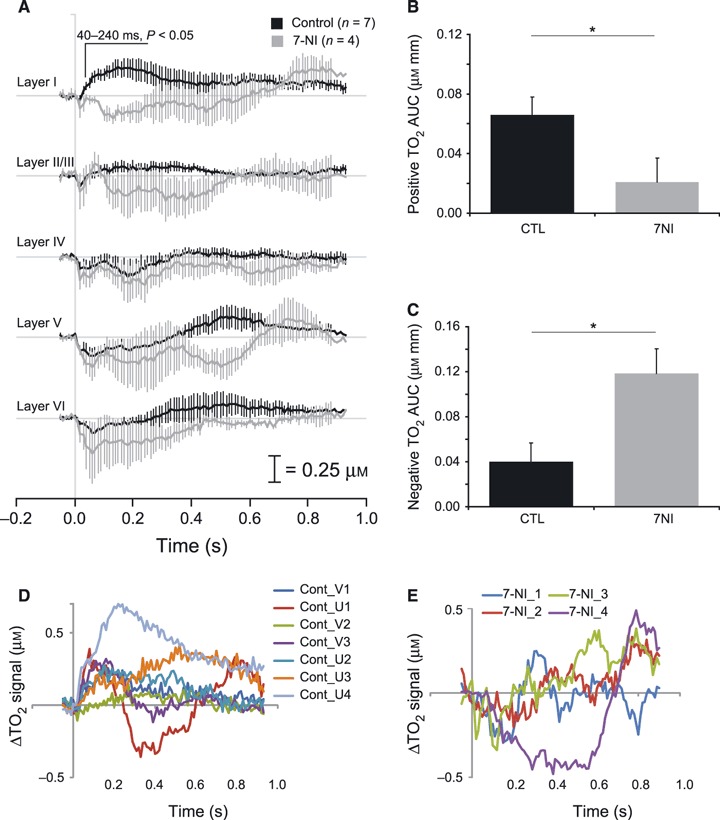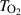 responses in control mice (*n* = 7) and mice treated with 7-NI (*n* = 4). (A) Mean 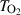 responses from control mice (black lines) and mice treated with 7-NI (grey lines). (B) Mean (+SEM) positive AUCs (0–500 ms) from layers I and II/III in control (CTL) mice (black bar) and mice treated with 7-NI (grey bar). (C). Mean (+SEM) negative AUCs (0–900 ms) from layers I-VI in control mice (black bar) and mice treated with 7-NI (grey bar). *P < 0.05. 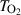 responses to whisker stimulation in layer I from individual mice (D, controls, *n* = 7; E, 7-NI-treated, *n* = 4). 7-NI_1, 7-NI mouse no. 1; Cont_V1, vehicle control mouse no. 1; Cont_U1, uninjected control mouse no. 1, etc.

In contrast to LFPs, 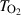 responses were very different in controls and 7-NI-treated mice. 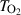 responses showed marked laminar differences in the control mice (Figs 11A and 12A), with a large positive 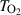 response in layer I (0.05 mm), a smaller but still positive 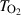 response in layer II/III (0.25 mm), a prominent negative 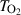 response with little overshoot in layer IV (0.45 mm), and a biphasic response (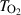 decrease then later overshoot) in layers V and VI (0.65 and 0.85 mm, respectively). This pattern of results was very similar to that seen in rats in Experiment 1. In contrast, responses in the 7-NI-treated mice were characterized by negative 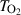 signals at all cortical depths, and a slow return to baseline with little or no overshoot, on average (Figs 11B and 12A). At most depths, negative 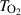 responses were larger in the 7-NI group than controls. 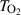 responses in layer I from individual control and 7-NI-treated mice are shown in Fig. 12D,E. [Note that in layer IV and V in both groups, there seemed to be a ‘double initial dip’. This may have been caused by additional movement of the cannula (and hence the whiskers contained therein) at the end of its travel.]

#### 7-nitroindazole decreases positive tissue O_2_ area under the curve but increases negative tissue O_2_ area under the curve

To analyse differences between 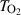 responses in the 7-NI and control groups, we calculated positive and negative AUCs. For the positive AUC we included responses from layers I and II/III (i.e. where positive 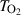 responses were prominent in the control group) and calculated the AUC over 500 ms after stimulus onset. This analysis (anova: drug treatment_2_ × cortical depth_2_ × *S*_11_) found a significant effect of drug treatment (*F*_1,9_ = 5.2; *P* = 0.048), with larger positive AUCs in the control group (Fig. 12B). There was no effect of cortical depth or interaction (*F*_s_ < 1.1; *P* > 0.3). For the negative AUC we included all cortical layers. When the negative AUC was calculated for the 500 ms after stimulus onset (anova: drug treatment_2_ × cortical depth_5_ × *S*_11_), there were no significant effects of drug, cortical depth, or interaction (*F*_s_ < 3.1; *P* > 0.1). However, when the AUC was calculated over 900 ms after stimulus onset there was a significant effect of drug condition (*F*_1,9_ = 8.1; *P* = 0.02), with higher negative AUCs in the 7-NI group (Fig. 12C). Thus, 7-NI treatment resulted in a decreased positive 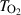 response and an increased negative 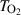 response.

#### No effect of 7-nitroindazole on slope of tissue O_2_ decrease

Importantly, there were no differences between the control and 7-NI groups in the rate of O_2_ consumption, as indexed by the slope of the initial 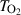 decrease. Analysis of the slope function for 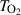 responses in layers IV, V, and VI (anova: drug treatment_2_ × cortical depth_3_ × *S*_11_) revealed no effect of drug treatment or interaction between drug treatment and cortical depth (all *F*_s_ < 1; *P* > 0.4). In addition, the correlation between LFP amplitude and negative 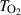 amplitude was almost identical in both groups (controls: *r* = 0.37; 7-NI: *r* = 0.39). Consistent with the findings of other groups (e.g. Offenhauser *et al.*, 2005), this shows that inhibition of nNOS does not affect the rate of 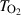 consumption.

#### Positive tissue O_2_ responses occur within 50 ms of stimulus onset

**Fig. 13:**
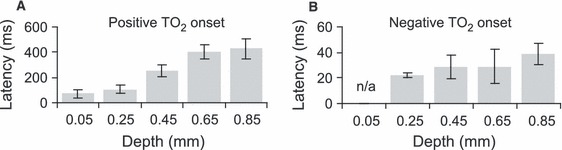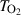 onset latencies (i.e. signal change greater than two SDs of baseline fluctuations) in control mice. Onset times for (A) positive and (B) negative 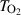 responses.

Previous studies have suggested that the positive 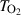 response is slow, of at least the order of several hundred milliseconds after stimulus onset, but the present data suggest that faster positive 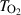 responses can occur. To quantify the latency of the positive 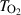 response in layer I (see Fig. 12A, top panel), we divided the 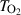 signal into 10 ms timebins (covering the 50 ms before stimulus onset and the 500 ms after stimulus onset) and compared 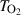 responses from the 7-NI and control groups (anova: drug treatment_2_ × timebin_56_ × *S*_11_). There was a main effect of drug treatment (*F*_1,9_ = 6.3; *P* = 0.03), with higher 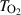 signals in the controls, and a drug treatment × timebin interaction (*F*_55,495_ = 2.3; *P* = 0.001). The interaction revealed no differences between the control and 7-NI groups during the baseline period but significantly higher 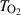 levels in the control group from 40 to 240 ms after stimulus onset. We further analysed onset latencies in control mice by calculating signal changes greater than two SDs of baseline fluctuations (Fig. 13), as was done in Experiment 1. Negative 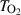 responses were not consistently observed in layer I and there were no differences in onset between the other layers (*F*_3,12_ = 1.7; *P* = 0.2). Positive 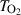 onset latencies were lowest in layer I and increased monotonically with depth. The mean positive 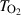 onset latency in layer I (0.05 mm) was 72 ± 30 ms, although this was positively skewed by one mouse with a high latency (249 ms) and the median latency was 50 ms. There was a main effect of cortical depth on positive 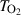 onset (*F*_4,15_ = 8.6; *P* = 0.001), driven by the lower latency in layer I compared with the other layers. These data demonstrate that positive 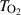 responses can occur within tens rather than hundreds of milliseconds.

## Discussion

### Summary of results

The present data show that changes in 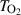 occur rapidly following neuronal activity, with evidence of both decreases and increases in 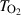 within 50 ms of stimulus onset. In addition, 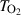 responses were laminar specific, with 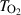 increases predominating in the superficial layers and 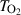 decreases predominating in the deeper layers. The largest decreases in 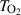 occurred in layer IV, i.e. in the same layer that the LFP amplitude was largest. Across layers, and when varying stimulus intensity within layer IV, LFP amplitude was a reliable predictor of the amplitude of the negative 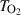 response but not the positive 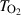 response. Moreover, our data show that the rapid, positive 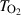 response is abolished following the inhibition of nNOS. Collectively, these data show that (i) contrary to current opinion, 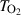 changes, both positive and negative, occur on a similar timescale to neuronal activity, i.e. in tens rather than hundreds or thousands of milliseconds; and (ii) there are strong laminar differences in 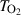 responses, probably reflecting laminar differences in local metabolic conditions and vascular architecture as well as differences in timing and degree of neuronal activity levels between cortical layers.

### Temporal dynamics of tissue O_2_ responses

The rapid changes in 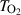 following whisker stimulation demonstrate the tight temporal coupling between neuronal and metabolic responses in the rat somatosensory cortex. 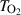 decreases were evident within 40 ms of stimulus onset, confirming the existence of an initial decrease in 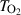 that is closely linked to the timing of neuronal activity. The initial 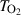 decrease has been reported in several previous studies (Ances *et al.*, 2001; Thompson *et al.*, 2003; Offenhauser *et al.*, 2005) but only at low temporal resolution (hundreds of milliseconds) and in response to sustained stimulation. Here, with much higher temporal resolution, we also show that it occurs rapidly and in response to single electrical pulses or mechanical deflections of the whiskers. However, during sustained stimulation, a slower-onset 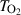 increase was observed (see Fig. 10), similar to that seen in previous studies (e.g. Ances *et al.*, 2001; Offenhauser *et al.*, 2005).

More surprisingly, we also observed increased 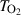 responses within 50 ms of stimulus onset when recording from the superficial layers. Such rapid 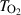 increases have never been reported previously and challenge current conceptions about the temporal limitations of 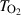 dynamics. It is possible that these changes reflect a small but rapid increase in CBF. Indeed, using laser–Doppler flowmetry, Neilsen and Laurtizen (2001) found increased CBF within 200 ms of stimulus onset in the superficial layers of the rat somatosensory cortex following stimulation of the infraorbital nerve. This response latency was at the temporal resolution limits of laser–Doppler flowmetry, leaving open the possibility that CBF might respond earlier than 200 ms under certain circumstances. Also, in the current study we found that the nNOS inhibitor, 7-NI, abolished positive 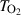 responses, which also implies a vascular basis.

However, we did not measure CBF directly in the present study and so cannot assert that this is the origin of the positive 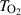 response. Moreover, there are alternative explanations for our findings that do not assume a change in CBF. Our rapid positive 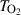 responses could occur under conditions where CBF remains constant but 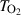 utilization decreases in the supragranular layers. For example, following the synchronous thalamic input to layer IV, excitatory activation spreads to the supragranular layers within a few milliseconds, accompanied by a substantial GABAergic activation (e.g. in layers II/III) that controls the spread of excitation within the barrel cortex (Petersen & Sakmann, 2001). It has previously been shown that increasing GABA_A_ activity (via local muscimol application) attenuates stimulus-induced 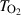 utilization (Caesar *et al.*, 2008). Thus, it could be that, during the fast positive 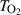 responses that we observed, CBF remains constant but there is a GABA_A_-mediated decrease in 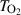 utilization in the supragranular layers that manifests as a net increase in 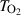. Future studies concomitantly measuring 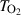 and CBF at high temporal resolution during manipulations of GABA_A_ activity are required to test this hypothesis. Even subtle disruption of neuronal-dependent vascular tone (e.g. reducing baseline blood flow) could interfere with this process. This could explain the abolition of the fast positive 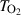 response seen in our 7-NI-treated mice in Experiment 2 without necessarily involving a drug effect on stimulus-induced changes in CBF.

### Relationship between local field potentials and tissue O_2_

There was a strong correlation between LFP amplitude and the amplitude of the initial 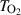 decrease, suggesting that not only are the two events closely linked in time but both can serve as an index of the strength of afferent input. This finding is consistent with previous studies. For example, following stimulation of the climbing fibre pathway in the rat cerebellum, Offenhauser *et al.* (2005) reported a high correlation between the sum of LFPs (i.e. ΣLFP = mean LFP amplitude × stimulation frequency) and the slope of the initial 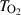 decrease. Moreover, blocking AMPA receptors (with topical application of the AMPA antagonist 6-cyano-7-nitroquinoxaline-2,3-dione) reduced both ΣLFPs and the size of the initial 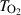 decrease. These data argue that the size of the initial 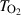 decrease is dependent upon glutamate release and excitatory post-synaptic activity. In the present study, LFP amplitude did not predict the size of the 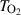 overshoot. Offenhauser *et al.* (2005) reported an exponential relationship between ΣLFP and positive 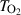 responses, such that ΣLFP did not predict positive 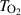 responses until a threshold of ΣLFP magnitude had been reached. In the present study, given that LFPs and 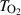 responses were evoked by single electrical pulses or whisker deflections, it is likely that this threshold was not reached, and hence no predictive relationship was found.

### Laminar nature of tissue O_2_ responses

The 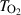 increases were seen in the superficial layers, whereas the 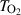 decreases were seen in the deeper layers. The largest 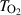 decreases were seen at the site of the largest LFPs, in layer IV, with smaller 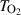 decreases observed dorsal and ventral to layer IV. This suggests that stimulus-induced 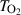 consumption in the rat barrel cortex reflects the magnitude of proximal excitatory post-synaptic activity. It is possible that the 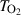 decrease could reflect a decrease in CBF but it is more commonly found that LFP amplitude (e.g. ΣLFP) correlates with increased rather than decreased CBF (Mathiesen *et al.*, 2000). This pattern of laminar-specific responses in 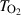 has not been reported before but our data are consistent with a recent fMRI study reporting whisker stimulation-induced positive BOLD responses in the superficial layers and negative BOLD responses in the deeper layers of the somatosensory cortex in alpha-chloralose-anaesthetized rats (Alonso *et al.*, 2008). Moreover, our data are also consistent with the distribution of cytochrome oxidase activity (a mitochondrial enzyme and sensitive marker for oxidative capacity), which shows dense staining of barrels in layer IV with less staining in the superficial layers (Anderson *et al.*, 2009). These data suggest that laminar differences in cellular organization may have important consequences for the size and direction of 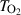 and BOLD responses, although it is important to note that laminar differences in vascular responses probably play a significant role (Cox *et al.*, 1993; Woolsey *et al.*, 1996; Tian *et al.*, 2010).

### Advantages and limitations of constant potential amperometry for tissue O_2_ measurements

The principal advantage of the technique used in the present study is its high temporal resolution and, as previously reported, its applicability in freely-moving rodents (Lowry *et al.*, 1997; McHugh *et al.*, 2011). In addition, although the CPE diameter is relatively large (125–250 μm) compared with some Clark-type O_2_ electrodes (Thompson *et al.*, 2003; Offenhauser *et al.*, 2005), CPEs can resolve laminar differences (∼200 μm) in the 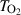 response and therefore have spatial resolution that is suitable for studying region-specific activation in rodents (McHugh *et al.*, 2011). Indeed, CPEs offer an advantage over smaller diameter electrodes in that there is a gradient of O_2_ tension from capillaries to mitochondria such that small O_2_ electrodes can detect widely varying O_2_ levels depending on their exact placement. In contrast, electrodes with dimensions larger than a capillary zone (i.e. >100 μm) detect an average level of 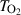 irrespective of placement.

Nevertheless, larger diameter electrodes do increase the possibility of tissue damage and/or tissue compression, but we do not think that these factors can account for our dataset for two reasons. First, depth recordings were counterbalanced across animals (i.e. dorsal-to-ventral in some animals, ventral-to-dorsal in others) and the results were the same irrespective of order. Second, reliable LFPs were seen in all recordings, indicating that, although some tissue damage may occur, the neurons in the vicinity of the electrode remain functional. Moreover, it is worth re-emphasizing that the rapid, laminar-specific 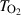 responses were observed in both Experiments 1 and 2 despite their methodological differences. In particular, the fact that similar results were obtained using different anaesthetics (urethane vs. halothane) and methods of ventilation (spontaneous breathing vs. artificial ventilation with O_2_/N_2_O) argues that these findings are not due to elevated or depressed levels of systemic oxygenation.

### Using the tissue O_2_ signal as a proxy for the blood-oxygen-level-dependent signal

Although the present study was in anaesthetized animals, our principal reason for developing this technique is to use the 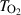 signal in freely-moving rodents to investigate region-specific brain activity during behavioural tasks (e.g. McHugh *et al.*, 2011). BOLD fMRI cannot be used in behaving rodents (because the subject’s head must remain stationary) but 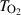 offers a good proxy measure for BOLD because both signals are driven by the same physiological mechanisms and respond in a similar fashion to reflect the balance of local O_2_ delivery and consumption (e.g. increased signals when CBF > O_2_ consumption; decreased signals when O_2_ consumption > CBF). Moreover, there is close concordance between the BOLD and 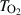 signals when recorded simultaneously in the fMRI scanner (Lowry *et al.*, 2010). Thus, 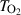 amperometry has the potential to improve translation between rodent and human studies across many areas of neuroscience.

## Conclusions

The relationships between neuronal, metabolic, and vascular responses are complex and understanding them requires mapping each signal at an appropriate level of spatial and temporal resolution. The present study has advanced this cause by demonstrating that changes in 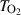 occur rapidly following neuronal activity and that the size and direction of these 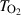 changes are laminar specific.

## Abbreviations

AUC: area under the curve
BOLD: blood-oxygen-level-dependent
CBF: cerebral blood flow
CPE: carbon paste electrode
fMRI: functional magnetic resonance imaging
LFP: local field potential
7-NI: 7-nitroindazole
nNOS: neuronal nitric oxide synthase
tissue oxygen (partial pressure of tissue oxygen)

## Supplementary material

Fig. S1. *In vitro* control data performed in aphosphate-buffered saline-filled glass cell showing no response ofthe constant potential amperometry O_2_ signal to a train of electrical stimulations.

As a service to our authors and readers, this journal provides supporting information supplied by the authors. Such materials are peer-reviewed and may be re-organized for online delivery, but are not copy-edited or typeset by Wiley-Blackwell. Technical support issues arising from supporting information (other than missing files) should be addressed to the authors.
